# Occlusion of the pulmonary artery by a primary pulmonary artery sarcoma resulting in cardiac arrest: a case report

**DOI:** 10.1186/s40981-019-0235-0

**Published:** 2019-02-28

**Authors:** Keitaro Tachi, Shinichi Inomata, Makoto Tanaka

**Affiliations:** Department of Anesthesiology, Division of Clinical Medicine, Faculty of Medicine, University of Tsukuba, Tsukuba, Ibaraki Japan

**Keywords:** Primary pulmonary artery sarcoma, Circulatory collapse, Extracorporeal circulation, Perioperative complications

## Abstract

**Background:**

Primary pulmonary arterial sarcoma (PPAS) is a rare condition. Although resection is recommended to improve prognosis, optimal anesthesia management for these cases remains unclear.

**Case presentation:**

A 62-year-old woman with a diagnosis of left pulmonary PPAS underwent surgical tumor resection and left lung pneumonectomy. Preoperative symptoms included a cough and hemoptysis. Computed tomography revealed a complete obstruction of the left pulmonary artery, with tumor extension into the right pulmonary artery, and mild tricuspid regurgitation was observed on the echocardiogram. Ninety minutes after anesthesia induction, the patient went into cardiopulmonary arrest. As the surgical field was sterilized, we proceeded with emergent sternotomy and cardiac massage. Extracorporeal circulation was established, and surgery proceeded once spontaneous circulation was recovered. The patient survived without neurological complications.

**Conclusions:**

Based on our experience and in the absence of evidence-based guidelines, the femoral artery and vein should be cannulated in all cases for extracorporeal circulation initiation before anesthesia induction.

## Background

Primary pulmonary artery sarcoma (PPAS) is a rare tumor that is associated with a poor prognosis. Complete surgical resection can improve the prognosis [1], although few studies have reported on the surgical management for PPAS [2, 3]. PPAS is associated with a risk of circulatory collapse in cases with increased central venous and right ventricular pressure [2]. We describe the case of a patient who went into cardiopulmonary arrest 90 min after anesthesia induction for complete resection of a PPAS. An emergency midline sternotomy was performed to establish extracorporeal circulation (ECC), and the patient was resuscitated. To our knowledge, this is the first report of a patient, without right heart failure, experiencing cardiac arrest after anesthesia induction because of an embolism caused by a change in the position of the tip of the PPAS. As part of our report, we describe the possible cause of circulatory collapse in this patient and a strategy for inducing anesthesia for patients with PPAS without remarkable right heart overload.

## Case presentation

**Fig. 1:**
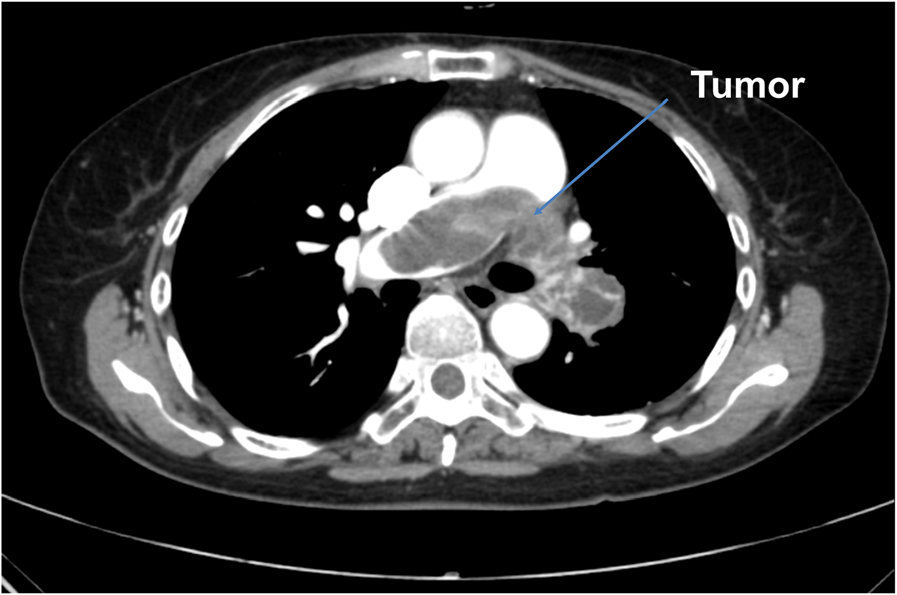
A tumor in the pulmonary artery was observed on chest computed tomography imaging. The mass filled the left pulmonary artery, from its distal portion and extended to the right pulmonary artery, which had minimal blood flow

The patient was a 62-year-old woman who was referred to our institution for surgical management of a suspected PPAS that was detected by enhanced computed tomography (CT). She had experienced hemoptysis and cough approximately once a week but had been able to continue her daily walks of about 4 km. There was no overswelling of the jugular vein or edema of the lower limbs in the physical examination of the patient. The results of biochemical blood tests were as follows: aspartate aminotransferase 18 U/l, alanine aminotransferase 11 U/l, gamma-glutamyl transpeptidase 15 U/l, and brain natriuretic peptide 30.6 pg/ml. The hepatic function tests were normal values and hepatic congestion was negative. Brain natriuretic peptide was also within normal limit. The chest CT revealed that the mass obstructed the trunk of the distal portion of the left pulmonary artery, extending into the right pulmonary artery (Fig. 1). The results of a blood gas analysis (at room air) were as follows: pH, 7.413; PaCO_2_, 41.1 mmHg; PaO_2_, 74.2 mmHg; hydrogencarbonate, 25.7 mmol/l; and base excess, 1.5 mmol/l. Tricuspid regurgitation (1°–2°) was identified on transthoracic echocardiogram. The right ventricular systolic pressure, estimated from tricuspid regurgitation, was 47 mmHg. Right heart strain was suspected based on this information. There was no enlargement of the inferior vena cava size at the end of expiration. There were no right ventricular hypertrophy/enlargement findings or displacement of the ventricular septum to the left ventricle side. There was no expansion of the right atrium. Given the above information, we determined that there were no remarkable right heart load findings.

The patient’s left ventricular ejection fraction, calculated using the modified Simpson’s method, was 70%, and there was no valve anomaly other than tricuspid regurgitation. The lumen of the right pulmonary artery was constricted, and a mosaic blood flow pattern was observed. From these findings, the patient was considered at risk of sudden death from right pulmonary artery occlusion and was scheduled for surgery.

Our surgical plan was to first perform the procedure for the pulmonary artery with the patient in the supine position, under ECC, followed by weaning from the ECC and moving the patient from the supine to the lateral decubitus position to perform the left lung pneumonectomy, via video-assisted thoracic surgery (VATS). The anesthesiologist strongly suggested inducing anesthesia after establishing ECC, using local anesthesia on the femoral artery and femoral vein. However, based on his experience, the surgeon did not deem it necessary.

The patient’s vital signs at the time of arrival in the operating theater were as follows: blood pressure, 147/81 mmHg; heart rate, 76 bpm; and SpO_2_, 100% (room air). An arterial pressure cannula was inserted into the left radial artery before anesthesia induction. Anesthesia was induced using fentanyl (50 μg), propofol (3 μg ml^−1^, target-controlled infusion), remifentanil (0.2 μg kg^−1^ min^−1^), and rocuronium (40 mg). The patient was intubated using a 32-Fr right-sided double-lumen tube (DLT) inserted into the right main bronchus. A transesophageal echocardiography (TEE) probe was inserted, as well as a central venous catheter, into the right internal jugular vein, with an initial central venous pressure (CVP) of 12 mmHg recorded. Anesthesia was carefully induced, and 43 min elapsed. As the location of the tip of the DLT could not be confirmed due to difficulty in passing the bronchofiberscope through the DLT, an attempt was made to re-intubate using a 35-Fr DLT. However, despite several attempts, the 35-Fr DLT could not be navigated beyond the patient’s glottis. We considered using a bronchial blocker. However, DLT is easier for one-lung ventilation and more useful for protecting the right lung against bleeding from the left lung during systemic heparinization. In addition, inserting a bronchial blocker into the left bronchus would hinder the surgical procedure of the left pneumonectomy, and separating lung ventilation could create instability by pulling on the left bronchus during surgery. For these reasons, we switched to a 32-Fr DLT and achieved successful intubation, although with a delay of 32 min from anesthesia induction.

After the induction of anesthesia, the patient was placed in the supine position for the first stage of the surgery.

**Fig. 2:**
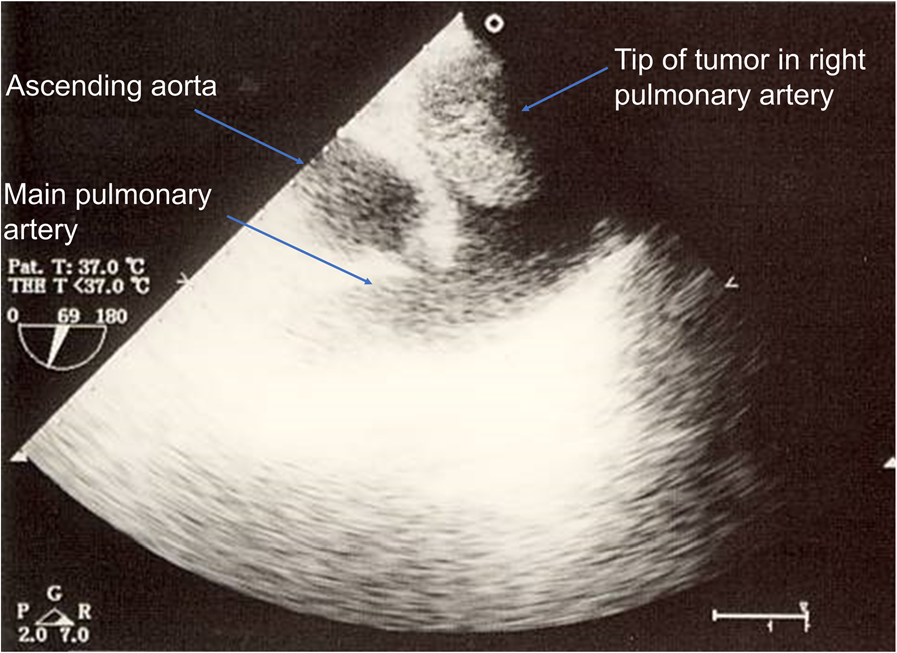
The tumor in the pulmonary artery was confirmed on intraoperative transesophageal echocardiography. The transesophageal echocardiography revealed a mobile mass, extending from the left main pulmonary artery (PA) to the right PA, which obstructed the lumen of the right PA (as shown on the mid-esophageal aortic valve short-axis view)

A pad was placed under the patient’s upper back to project the chest forward and to extend the neck, and the surgical site was sterilized. The bronchofiberscope available in the operating room did not pass through the 32-Fr DLT. Considering, again, that the location of the tip of the DLT could not be confirmed, a right one-lung ventilation (OLV) was implemented on a trial basis. Once the OLV was established, cardiac function and the tumor location were evaluated using TEE. During TEE monitoring, a movable tumor was observed between the left and right pulmonary arteries (Fig. 2). This procedure required 15 min.

**Fig. 3:**
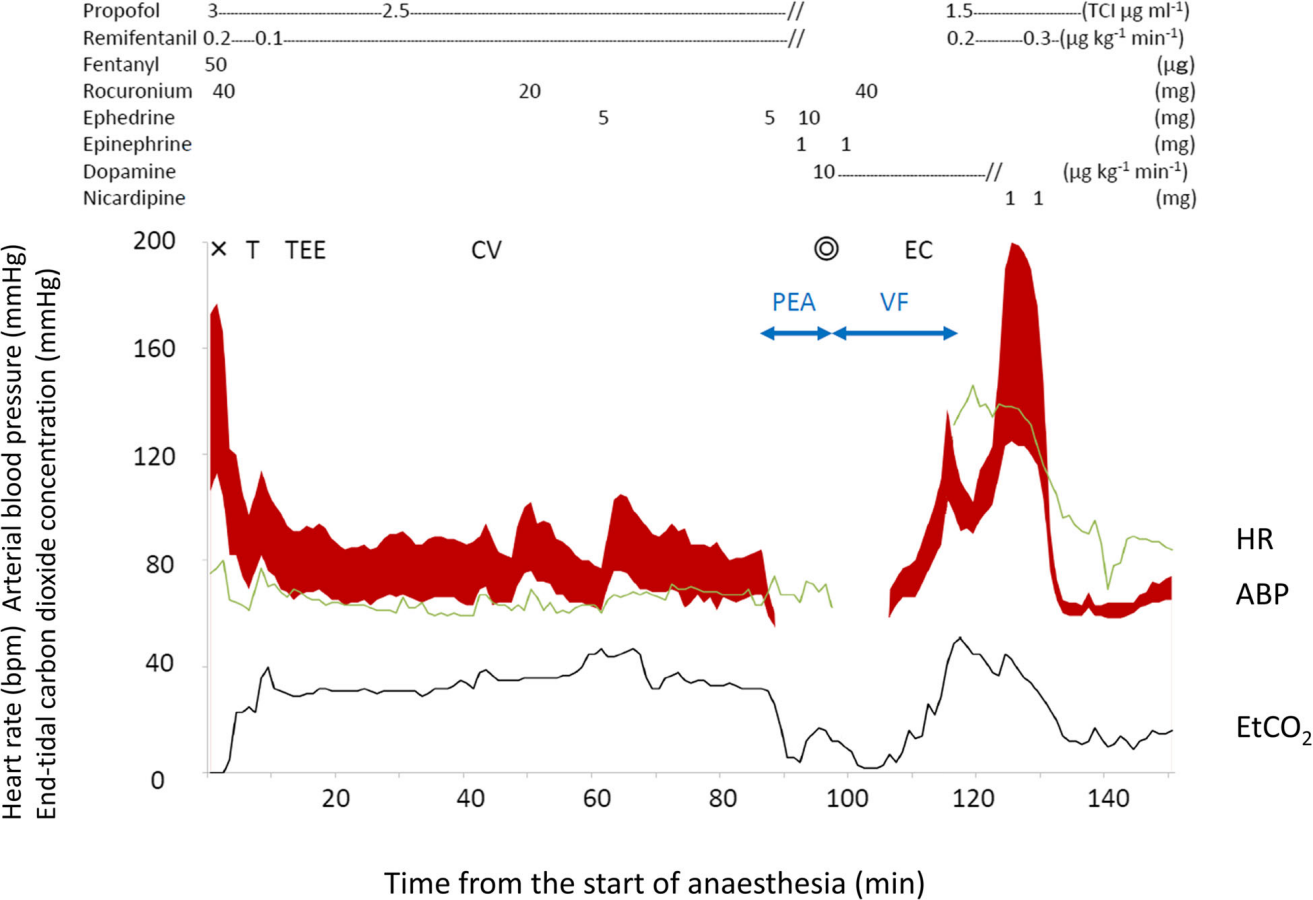
Cardiopulmonary events under anesthesia. The patient went into cardiopulmonary arrest 90 min after induction of general anesthesia. The surgical procedure was initiated while cardiac compressions were being performed and cardiopulmonary bypass was established, with recovery of the cardiac rhythm at 25 min after cardiopulmonary arrest. The horizontal axis of the graph represents the time (min) after induction of anesthesia, with the following variables plotted on the vertical axis: arterial blood pressure (ABP: red band graph [mmHg]), heart rate (HR: green line [bpm]), and end-tidal carbon dioxide concentration (EtCO_2_: gray line [mmHg]). Multiplication sign: induction of anesthesia; T: tracheal intubation; TEE: insertion of transesophageal echocardiography probe; CV: insertion of central venous catheter; bullseye symbol: start of the surgery; EC: establishment of extracorporeal circulation; PEA: pulseless electrical activity; VF: ventricular fibrillation.

The anesthesia record is shown in Fig. 3. At 90 min after anesthesia induction, the patient’s arterial pressure suddenly dropped to 0 mmHg, with TEE indicating a marked low volume of blood in the left ventricular cavity. The electrocardiogram revealed that the patient was in sinus bradycardia.

Propofol and remifentanil, which had been continuously administered to the patient, were discontinued. Ephedrine (15 mg) and phenylephrine (100 μg) were administered under rapid fluid infusion. This management was ineffective, and we immediately began chest compressions. As the right OLV had just been established and TEE started, we suspected retraction of the pulmonary artery (caused by the right OLV or the TEE probe) as the cause of the patient’s cardiac arrest. The TEE was removed and the right OLV was terminated. However, the patient’s blood pressure did not increase. A median sternotomy was performed, without ECC, and ventricular fibrillation occurred during the procedure. We administered two 1-mg doses of epinephrine and proceeded with the surgical procedure, without performing direct current defibrillation. Immediately, after the pericardial incision was completed, the heart was massaged directly and ECC (inflow from the right atrium and outflow to the ascending aorta) was established, 21 min after the time of cardiac arrest. Cardiac rhythm was recovered, with 25 min as the total time from cardiac arrest to the re-establishment of spontaneous circulation (Fig. 3).

**Fig. 4:**
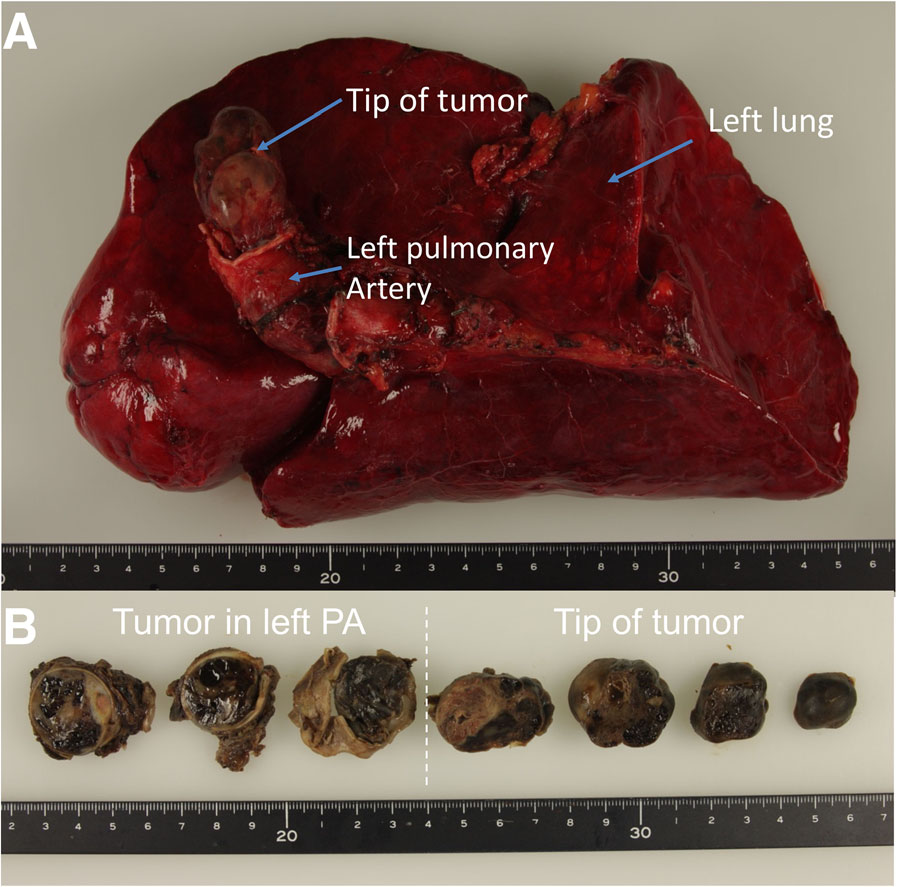
**a** Pathological specimen, including the tumor, the left pulmonary artery (PA), and the left lung. **b** The cross section of the tumor was 1 cm in thickness. The mass protruded about 3 cm into the right PA and filled the left PA

Continuous propofol administration was resumed at 1.5 μg ml^−1^ (target-controlled infusion) and remifentanil at 0.2 μg kg^−1^ min^−1^_,_ and we were able to subsequently proceed with the planned surgery. The left pulmonary artery was completely separated from the tumor and the main pulmonary artery, with the resultant defect closed with a patch. The tumor was a smooth-surfaced elastic mobile mass (Fig. 4a, b), which was adherent to the right pulmonary artery, approximately 3 cm from the bifurcation of the pulmonary artery (Fig. 4a, b). A left lung pneumonectomy was performed after weaning from ECC. Prior to surgery, we had planned to perform VATS with the patient in the lateral decubitus position. However, as bleeding persisted and vital signs were not stabilized, a change in the position was not a feasible option, and we proceeded with the surgical procedure with the patient in the supine position with chest projection. The left lung was congested, hard, and swollen, and it did not collapse with right OLV. We deemed that this congestion of the left lung was likely caused by backflow from the pulmonary vein or bronchial arteries during the ECC management. As such, VATS was not possible and the procedure was changed to an open procedure, via a fourth intercostal thoracotomy. The TEE probe was reinserted during ECC. The wall motion of the heart was maintained during the procedure, with no change in the degree tricuspid regurgitation. Anesthesia was maintained using propofol (1.5–2 μg ml^−1^, target-controlled infusion), remifentanil (0.2–0.3 μg kg^−1^ min^−1^) and rocuronium (30 mg h^−1^). The patient received a bolus administration of phenylephrine (100 μg) several times, with continuous administration of dopamine (5–8 μg kg^−1^ min^−1^) and noradrenaline (0.3 μg kg^−1^ min^−1^), to manage the persistent hypotension. Nitroglycerin (0.5 μg kg^−1^ min^−1^) was administered to lower the patient’s pulmonary vascular resistance and cardiac protection. The patient’s head was cooled using ice packs. Operation time was 9 h 52 min, ECC management time was 3 h 1 min, anesthesia time was 12 h 22 min, and the bleeding volume was 5890 ml. Of note, a large amount of bleeding occurred during the left pneumonectomy procedure. For this reason, a massive infusion (21,900 ml) and a massive transfusion (7680 ml) were required during the surgery. The total left pneumonectomy and massive transfusion resulted in a protracted timeline for ventilator weaning, with the patient extubated 14 days after surgery. After extubation, the patient required noninvasive positive-pressure ventilation. The patient was gradually weaned from supportive ventilation and was discharged from the intensive care unit 20 days after surgery. After discharge, the patient experienced temporary left recurrent nerve palsy, articulation disorder, and motor disorder in hand function. The patient’s condition recovered gradually, and she was discharged from the hospital 60 days after surgery. Seven months after the surgery, the patient was able to walk for 30 min without assistance. The pathological diagnosis confirmed an intimal sarcoma of the left pulmonary artery.

## Discussion

PPAS is a rare tumor, first documented in 1923 [4]. Since then, approximately 400 cases of PPAS have been reported [1]. In the absence of specific symptoms, early diagnosis is difficult [1], although advances in imaging have improved detection. The tumor grows and fills the pulmonary artery lumen, with the obstruction causing dyspnea, pulmonary hypertension, and elevation of right ventricular pressure. Without treatment, the prognosis is poor, with a life expectancy of approximately 6 weeks after diagnosis, and death caused by bilateral pulmonary artery occlusion [5–7].

In a review of 391 cases of PPAS [1], patients (155 cases, 44%) who underwent complete resection had improved prognoses, compared to those who underwent partial resection or no treatment. Various chemotherapy and radiotherapy regimens have been used but no definitive regimens have been established, and there is no evidence of an advantage of chemotherapy or radiotherapy over surgery [1, 5–11]. Therefore, complete surgical resection is the best alternative to prevent sudden death and improve outcome. Advancement in imaging-based diagnosis has improved early detection of PPAS. Early surgery, diagnosis confirmation, and multimodal therapy have also improved the prognosis of PPAS [1, 11]. For bilateral PPAS, it is necessary to perform a pulmonary artery endarterectomy. This procedure requires deep hypothermia and circulation arrest to prevent backflow from bronchial arteries into the pulmonary artery [3]. For unilateral PPAS, diagnosed early, a pneumonectomy is recommended. Overall, the prognosis is better for pneumonectomy than pulmonary artery endarterectomy [12, 13].

In a case series of 93 patients with PPAS, the reported perioperative mortality rate of PPAS was high at 22% [5]. Anesthesia management for PPAS surgery is challenging. In our PubMed search, using “pulmonary artery sarcoma” and “anesthesia” as keywords, we identified only two case reports describing anesthesia management for PPAS surgery [2, 3]. Clear guidelines have yet to be established. Pulmonary thromboembolism (PTE), caused by peripheral thrombus, closely presents as PPAS; therefore, some of the methods of anesthesia for PTE can be applied to the management of PPAS. There is a 19% incidence rate of circulatory collapse after induction of general anesthesia during thrombectomy for acute PTE, but no clear predictive factors of this complication have been identified [14]. In their report on PPAS, Flexman et al. [2] reported findings of right heart overload prior to surgery, including dyspnea and a marked increase in central venous pressure (CVP) and right ventricular pressure. In our case, because of circulatory collapse after induction of general anesthesia, emergent median sternotomy was necessary to start ECC. In contrast, in the PPAS case report published by Hoogma et al. [3], an increase in CVP or findings of right heart overload was not identified, and there was no mention of deterioration in circulation dynamics after induction of anesthesia.

Circulatory collapse after induction of anesthesia has not been reported in patients with PPAS in the absence of remarkable right heart failure prior to surgery. To our knowledge, this is the first report of cardiac arrest caused by an embolism due to a change in the position of the tip of the PPAS in the perioperative period. Advancement in imaging-based diagnosis has improved early detection of PPAS. Similar to our case, there will likely be more cases of PPAS without findings of right heart overload prior to surgery because early surgery and diagnosis of PPAS improve prognosis [1]. Given this case, we should consider that there could be a risk of circulatory collapse during induction of anesthesia despite not finding right heart overload in the perioperative examination.

In our case, cardiopulmonary arrest was likely due to two potential causes. First, owing to its gelatinous properties [9, 10, 15–17], the tumor moved during the procedure and extended within the lumen of the pulmonary artery, as a result of the slight change in body position on the surgical table. An intimal sarcoma (angiosarcoma) is a rare tumor, identified in 7% of PPAS cases [1] and, because of its soft, pliable properties, the tumor can fill the vessel lumen [9, 10]. This gelatinous property of a PPAS is different from that of a thrombus. A previous study has, in fact, demonstrated the “to-and-fro” movement of a PPAS within the right pulmonary artery on arterial angiography, while still maintaining normal blood flow [18]. Unlike PTE, a PPAS is characterized by the main pulmonary artery serving as the site of the primary lesion, and is soft, elastic, and mobile [9, 10, 15–17]. Although the tumor does not originally occlude the pulmonary artery, migration and spread of the tumor can possibly cause occlusion of the pulmonary artery, with a consequent abrupt change in hemodynamics.

Second, the pulmonary artery may also have been mechanically compressed within the mediastinum because of the DLT and TEE probe. In our case, changes in intrathoracic pressure due to the start of the right OLV may have influenced pulmonary arterial blood flow. On CT, we identified the tumor in the pulmonary artery, with the trachea and the esophagus being close in proximity. We considered that TEE, DLT, and OLV contributed to cardiac arrest, indirectly but additively. Upon intraoperative observation, the tip of the tumor was trapped in the right pulmonary artery. Following the abrupt decline in blood pressure, the patient went into cardiac arrest due to the irreversible disruption in the blood flow. This would explain the lack of improvement in blood pressure after removal of the TEE and termination of the OLV. Although there is a possibility of influencing the hemodynamics, as described above, using a DLT is essential to maintain ventilation in cases requiring unilateral pneumonectomy. Moreover, as there is a risk of tumor fragmentation, TEE is necessary for continuous monitoring of the tumor location. In addition, as a pulmonary artery catheter is difficult to use, TEE can further be used to monitor cardiac function.

Induction of general anesthesia during surgery for PPAS should include the following: the femoral artery and vein should always be cannulated, with preparation of a femoral-femoral (F-F) bypass, under local anesthesia and prior to induction of general anesthesia, even in cases without presurgical right heart overload or unilateral tumor, as well as in patients with tumors in the main pulmonary artery trunk, from the right ventricle outflow tract. Unlike acute PTE, with PPAS, there is no risk of further deterioration of pulmonary embolic symptoms because of a thrombus in the lower limb because of the F-F bypass [14].

In conclusion, we present the case of a patient with PPAS who did not present with findings of remarkable right heart overload before surgery but still went into cardiopulmonary arrest after anesthesia induction. With rapid resuscitation, the patient survived without neurological complications. Based on our experience, a F-F bypass should be initiated, or at least prepared, before anesthesia induction, in patients with a tumor near the main pulmonary artery trunk, even in the absence of right heart overload.

## Abbreviations

CT: Computed tomography
CVP: Central venous pressure
DLT: Double-lumen tube
ECC: Extracorporeal circulation
OLV: One-lung ventilation
PPAS: Primary pulmonary artery sarcoma
PTE: Pulmonary thromboembolism
TEE: Transesophageal echocardiography
VATS: Video-assisted thoracic surgery

## References

[1] Bandyopadhyay D, Panchabhai TS, Bajaj NS, Patil PD, Bunte MC (2016). Primary pulmonary artery sarcoma: a close associate of pulmonary embolism—20-year observational analysis. J Thorac Dis.

[2] Flexman AM, Del Vicario G, Schwarz SK (2009). Hemodynamic collapse under anesthesia in a patient with pulmonary artery sarcoma. Can J Anaesth.

[3] Hoogma D, Meyns B, Van Raemdonck D, Van de Velde M, Missant C, Rex S (2015). Anesthetic management for resection of bilateral pulmonary artery sarcoma. A Case Rep.

[4] Mandelstamm M (1923). Über primäre Neubildungen des Herzens. Virchows Arch Pathol Anat.

[5] Krüger I, Borowski A, Horst M, de Vivie ER, Theissen P, Gross-Fengels W (1990). Symptoms, diagnosis, and therapy of primary sarcomas of the pulmonary artery. Thorac Cardiovasc Surg.

[6] Cox JE, Chiles C, Aquino SL, Savage P, Oaks T (1997). Pulmonary artery sarcomas: a review of clinical and radiologic features. J Comput Assist Tomogr.

[7] Blackmon SH, Rice DC, Correa AM, Mehran R, Putnam JB, Smythe WR (2009). Management of primary pulmonary artery sarcomas. Ann Thorac Surg.

[8] Nakahira A, Ogino H, Sasaki H, Katakami N (2007). Long-term survival of a pulmonary artery sarcoma produced by aggressive surgical resection and adjuvant chemoradiotherapy. Eur J Cardiothorac Surg.

[9] Long HQ, Qin Q, Xie CH (2008). Response of pulmonary artery intimal sarcoma to surgery, radiotherapy and chemotherapy: a case report. J Med Case Rep.

[10] Hirose T, Ishikawa N, Hamada K, Inagaki T, Kusumoto S, Shirai T (2009). A case of intimal sarcoma of the pulmonary artery treated with chemoradiotherapy. Intern Med.

[11] Wong HH, Gounaris I, McCormack A, Berman M, Davidson D, Horan G (2015). Presentation and management of pulmonary artery sarcoma. Clin Sarcoma Res.

[12] Chhaya NC, Goodwin AT, Jenkins DP, Pepke-Zaba J, Dunning JJ (2006). Surgical treatment of pulmonary artery sarcoma. J Thorac Cardiovasc Surg.

[13] Grazioli V, Vistarini N, Morsolini M, Klersy C, Orlandoni G, Dore R (2014). Surgical treatment of primary pulmonary artery sarcoma. J Thorac Cardiovasc Surg.

[14] Rosenberger P, Shernan SK, Shekar PS, Tuli JK, Weissmüller T, Aranki SF (2006). Acute hemodynamic collapse after induction of general anesthesia for emergent pulmonary embolectomy. Anesth Analg.

[15] Kerr KM (2005). Pulmonary artery sarcoma masquerading as chronic thromboembolic pulmonary hypertension. Nat Clin Pract Cardiovasc Med.

[16] Ozbek C, Emrecan B, Calli AO, Gurbuz A (2007). Intimal sarcoma of the pulmonary artery with retrograde extension into the pulmonic valve and right ventricle. Tex Heart Inst J.

[17] Nakajima J, Morota T, Matsumoto J, Takazawa Y, Murakawa T, Fukami T (2007). Pulmonary intimal sarcoma treated by a left pneumonectomy with pulmonary arterioplasty under cardiopulmonary bypass: report of a case. Surg Today.

[18] Hynes JK, Smith HC, Holmes DR, Edwards WD, Evans TC, Orszulak TA (1982). Preoperative angiographic diagnosis of primary sarcoma of the pulmonary artery. Circulation.

